# The chromosomal genome sequence of the mollusc, *Ctena decussata *(O.G.Costa, 1829) and its bacterial endosymbiont *Candidatus* Thiodiazotropha sp. CDECU1 (Chromatiales)

**DOI:** 10.12688/wellcomeopenres.24738.1

**Published:** 2025-08-11

**Authors:** Laetitia Wilkins, Benedict Yuen, Jillian Petersen, Graeme Oatley, Elizabeth Sinclair, Eerik Aunin, Noah Gettle, Camilla Santos, Michael Paulini, Haoyu Niu, Victoria McKenna, Rebecca O’Brien, Wellcome Sanger Institute Tree of Life Management, Samples and Laboratory Team, Wellcome Sanger Institute Scientific Operations: Sequencing Operations, Wellcome Sanger Institute Tree of Life Core Informatics Team, EBI Aquatic Symbiosis Genomics Data Portal Team, Aquatic Symbiosis Genomics Project Leadership

**Affiliations:** 1 Max Planck Institute for Marine Microbiology, Bremen, Germany; 2 Centre for Microbiology and Environmental Systems Science, University of Vienna, Vienna, Austria; 3 Tree of Life, Wellcome Sanger Institute, Hinxton, England, UK

**Keywords:** Ctena decussata, Bivalvia, genome sequence, chromosomal, Lucinida; microbial metagenome assembly

## Abstract

We present a genome assembly from a specimen of *Ctena decussata* (Mollusca; Bivalvia; Lucinida; Lucinidae). The genome sequence has a total length of 1,658.05 megabases. Most of the assembly (97.83%) is scaffolded into 18 chromosomal pseudomolecules. The mitochondrial genome has also been assembled and is 53.28 kilobases in length. The genome of *Candidatus* Thiodiazotropha sp. CDECU1, a bacterium associated with *C. decussata* was also assembled,

## Species taxonomy

Eukaryota; Opisthokonta; Metazoa; Eumetazoa; Bilateria; Protostomia; Spiralia; Lophotrochozoa; Mollusca; Bivalvia; Autobranchia; Heteroconchia; Euheterodonta; Imparidentia; Lucinida; Lucinoidea; Lucinidae; *Ctena*; *Ctena decussata* (O.G.Costa, 1829) (NCBI:txid881212)

## Background

*Ctena decussata* was originally described as *Lucina decussata* by Costa (1829) and the genus name *Ctena* was introduced by Moerch (1860). The *Ctena* genus includes 14 recognised species. It belongs to the bivalve family Lucinidae, the members of which are characterised by their association with chemosynthetic endosymbiont bacteria that are housed within specialised bacteriocytes in the gills ( Taylor & Glover, 2021). *Ctena decussata* has a white shell that can become more yellowish towards the umbo and reaches approximately 20 mm in length. The outline of the shell is circular to ovate with the anterior-dorsal margin slightly concave. It has a short, shallow lunule and slightly longer shallow escutcheon. The shell is tumid and sculptured with both radial ribs and concentric lamellae with visible growth stages and crenulated margins. *Ctena decussata* is a sister species to *Ctena eburnea* but has finer and more numerous radial ribs ( Taylor *et al.*, 2022; Von Cosel & Gofas, 2019).

*Ctena decussata* is distributed in the eastern Atlantic, ranging from the Canary Islands and Madeira to northern Spain ( Oliver *et al.*, 2016), but is most abundant throughout the Mediterranean where it likely re-entered the sea during the early Pliocene following the Late Miocene desiccation event ( Taylor & Glover, 2021). Early records of this species in the British Isles from were considered adventitious ( Taylor *et al.*, 2013). It is often found burrowed in subtidal coarse to gravelly sand but may also occur in the thick rhizome stratum of *Posidonia oceanica* meadows and other seagrasses ( Holzknecht & Albano, 2022).

The genome of the intracellular bacterial symbionts hosted by *Ctena decussata* sampled on the French Mediterranean coast was recently sequenced. It shares an ANI greater than 96–98% with a symbiont of *Ctena orbiculata* from Florida, indicating that the *Ctena decussata* symbiont species has a large dispersal capability ( Lim *et al.*, 2019; Sudo *et al.*, 2024). This symbiont species shares similar metabolic capabilities to other previously described lucinid symbionts. These capabilities include nitrogen fixation, multiple pathways for oxidising reduced sulfur compounds, and the ability to fix inorganic carbon through the Calvin-Benson-Bassham pathway ( Petersen & Yuen, 2021). The metabolic functions of lucinid symbiosis may play a crucial ecological role in coastal ecosystems by contributing to nutrient cycling and supporting commercially important food chains (e.g. Chin *et al*., 2021; Higgs *et al*., 2016; Van Der Heide *et al*., 2012).

These genomic resources will facilitate the development of the lucinid symbiosis as an experimental system for studying animal-bacteria interactions. Furthermore, chemosymbiosis has arisen multiple times independently in different bivalve lineages ( Sogin *et al.*, 2021). These resources will also enable further studies investigating the genomic basis of the unique morphological, physiological, and behavioral adaptations that have accompanied the emergence of chemosymbiosis across different animal groups.

## Genome sequence report

### Sequencing data

**Figure 1.**
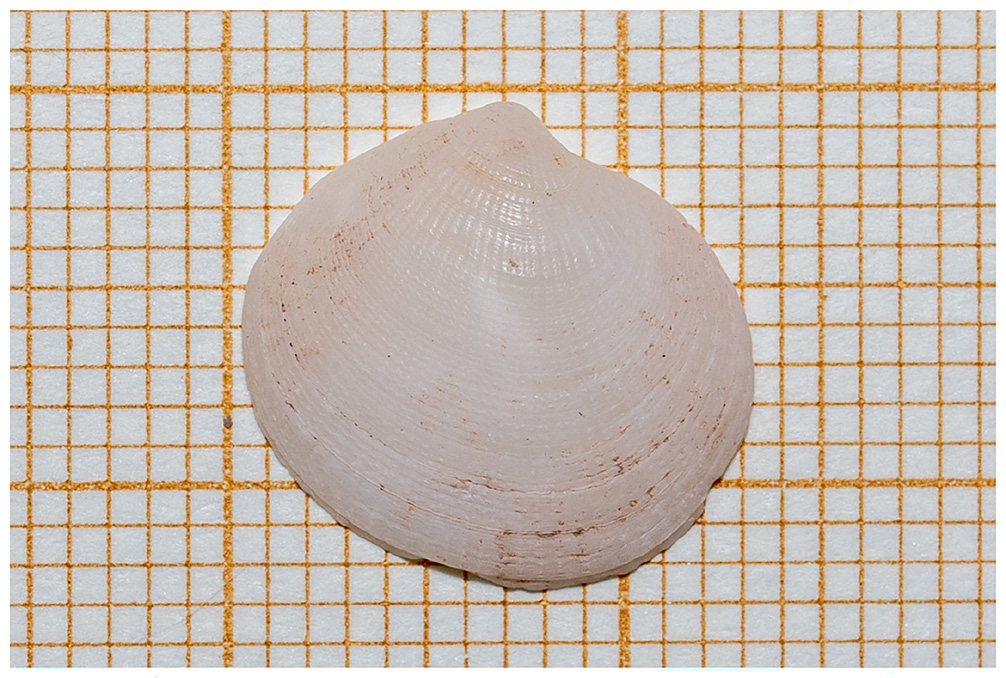
Photograph of *Ctena decussata* (Photograph by lizard--o_o on iNaturalist CC-BY-NC).

The genome of a specimen of *Ctena decussata* ( Figure 1) was sequenced using Pacific Biosciences single-molecule HiFi long reads, generating 92.81 Gb from 10.47 million reads. Based on the estimated genome size, the sequencing data provided approximately 50 coverage of the genome. Chromosome conformation Hi-C data produced 0.00 Gb from 0.00 million reads. RNA sequencing data were also generated and are available in public sequence repositories. Table 1 summarises the specimen and sequencing information.

**Table 1.** Specimen and sequencing data for *Ctena decussata*.

Project information			
**Study title**	Ctena decussata		
**Umbrella BioProject**	PRJEB65003		
**Species**	*Ctena decussata*		
**BioSpecimen**	SAMEA13422730		
**NCBI taxonomy ID**	881212		
Specimen information			
**Technology**	**ToLID**	**BioSample accession**	**Organism part**
**PacBio long read sequencing**	xbCteDecu1	SAMEA13422739	gill
**Hi-C sequencing**	xbCteDecu1	SAMEA13422741	muscle
**RNA sequencing**	xbCteDecu2	SAMEA13422742	gill
Sequencing information			
**Platform**	**Run accession**	**Read count**	**Base count (Gb)**
**PacBio Sequel IIe**	ERR11843469	2.17e+06	21.06
**PacBio Revio**	ERR11843468	8.30e+06	71.75
**RNA Illumina NovaSeq X**	ERR13669941	1.11e+08	16.82

### Assembly statistics

The primary haplotype was assembled, and contigs corresponding to an alternate haplotype were also deposited in INSDC databases. The assembly was improved by manual curation, which corrected 460 misjoins or missing joins and removed 243 haplotypic duplications. These interventions reduced the total assembly length by 13.66% and decreased the scaffold count by 40.0%. The final assembly has a total length of 1,658.05 Mb in 260 scaffolds, with 1,003 gaps, and a scaffold N50 of 92.3 Mb ( Table 2).

**Table 2.** Genome assembly data for *Ctena decussata*.

Genome assembly		
Assembly name	xbCteDecu1.1	
Assembly accession	GCA_963989385.1	
*Alternate haplotype accession*	*GCA_963989355.1*	
Assembly level for primary assembly	chromosome	
Span (Mb)	1,658.05	
Number of contigs	1,263	
Number of scaffolds	260	
Longest scaffold (Mb)	129.49	
Assembly metric	Measure	*Benchmark*
Contig N50 length	4.64 Mb	*≥ 1 Mb*
Scaffold N50 length	92.3 Mb	*= chromosome N50*
Consensus quality (QV)	Primary: 64.3; alternate: 62.7; combined 63.4	*≥ 40*
*k*-mer completeness	Primary: 56.97%; alternate: 57.87%; combined: 98.57%	*≥ 95%*
BUSCO *	C:80.6%[S:78.5%,D:2.0%],F:4.1%,M:15.3%,n:5,295	*S > 90%, D < 5%*
Percentage of assembly assigned to chromosomes	97.84%	*≥ 90%*
Organelles	Mitochondrial genome: 53.28 kb	*complete single alleles*

* BUSCO scores based on the mollusca_odb10 BUSCO set using version 5.5.0. C = complete [S = single copy, D = duplicated], F = fragmented, M = missing, n = number of orthologues in comparison.

**Figure 2.**
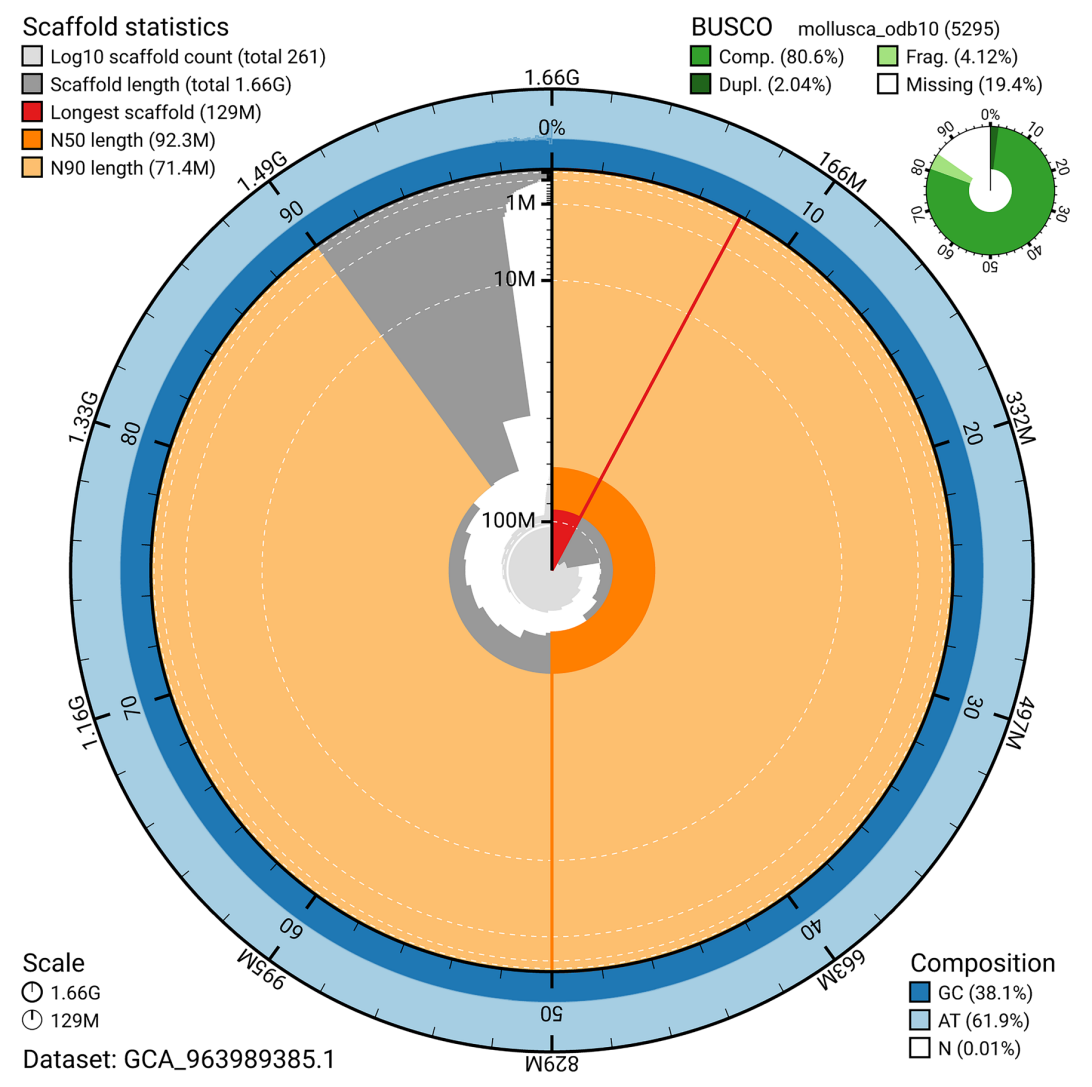
Genome assembly of *Ctena decussata*, xbCteDecu1.1: metrics. The BlobToolKit snail plot provides an overview of assembly metrics and BUSCO gene completeness. The circumference represents the length of the whole genome sequence, and the main plot is divided into 1,000 bins around the circumference. The outermost blue tracks display the distribution of GC, AT, and N percentages across the bins. Scaffolds are arranged clockwise from longest to shortest and are depicted in dark grey. The longest scaffold is indicated by the red arc, and the deeper orange and pale orange arcs represent the N50 and N90 lengths. A light grey spiral at the centre shows the cumulative scaffold count on a logarithmic scale. A summary of complete, fragmented, duplicated, and missing BUSCO genes in the mollusca_odb10 set is presented at the top right. An interactive version of this figure is available at https://blobtoolkit.genomehubs.org/view/GCA_963989385.1/dataset/GCA_963989385.1/snail.

**Figure 3.**
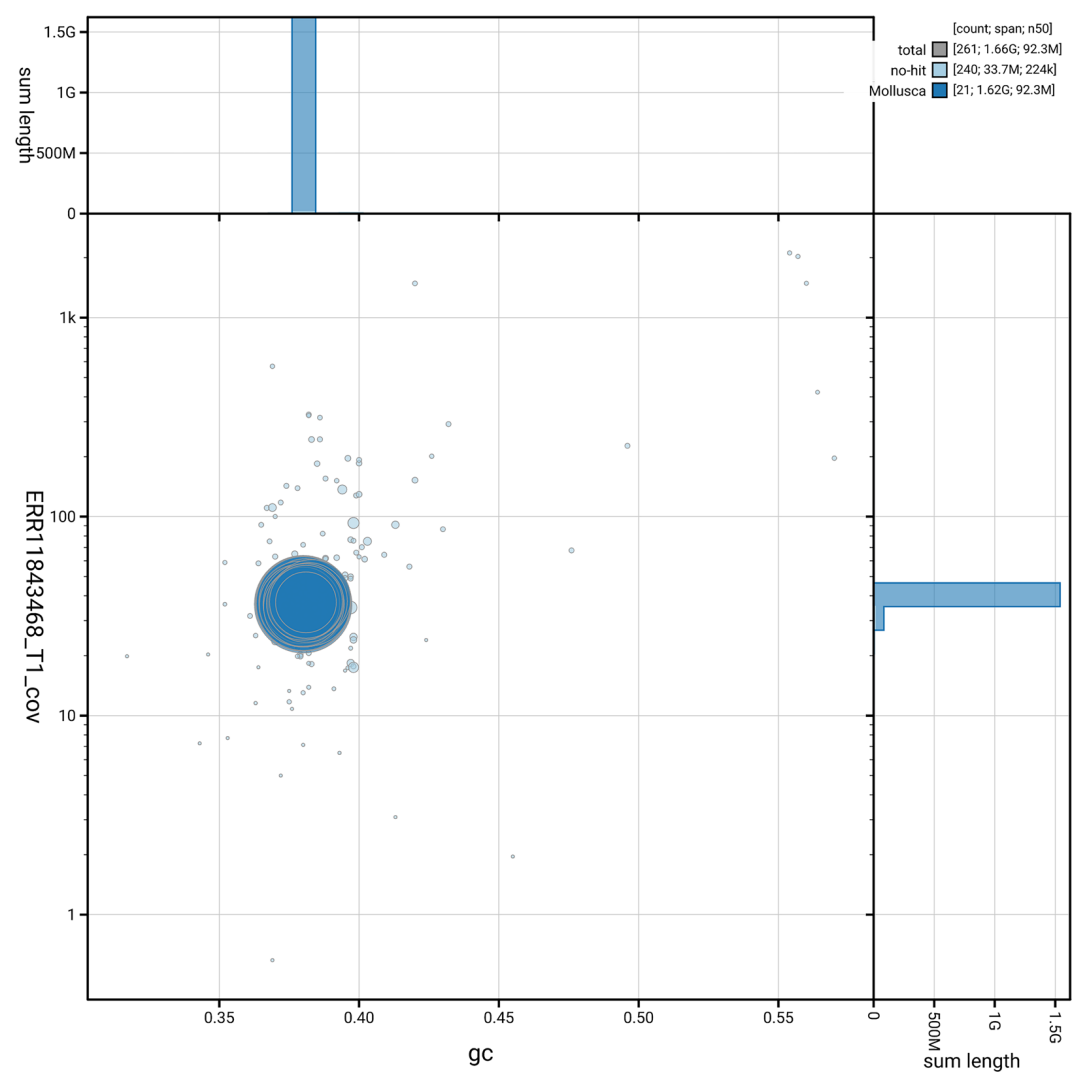
Genome assembly of *Ctena decussata*, xbCteDecu1.1: BlobToolKit GC-coverage plot. Blob plot showing sequence coverage (vertical axis) and GC content (horizontal axis). The circles represent scaffolds, with the size proportional to scaffold length and the colour representing phylum membership. The histograms along the axes display the total length of sequences distributed across different levels of coverage and GC content. An interactive version of this figure is available at https://blobtoolkit.genomehubs.org/view/GCA_963989385.1/dataset/GCA_963989385.1/blob.

**Figure 4.**
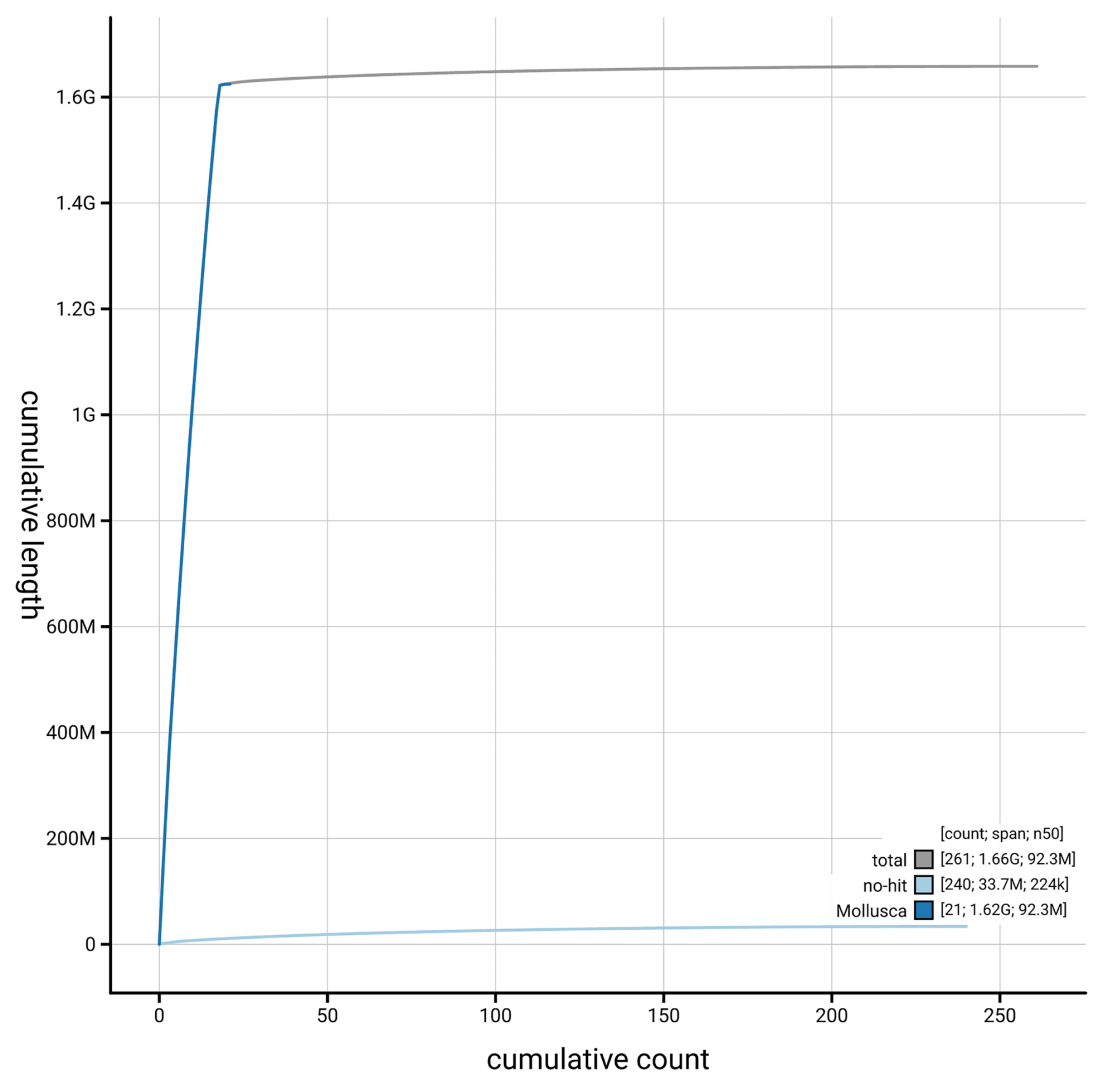
Genome assembly of *Ctena decussata,* xbCteDecu1.1: BlobToolKit cumulative sequence plot. The grey line shows cumulative length for all scaffolds. Coloured lines show cumulative lengths of scaffolds assigned to each phylum using the buscogenes taxrule. An interactive version of this figure is available at https://blobtoolkit.genomehubs.org/view/GCA_963989385.1/dataset/GCA_963989385.1/cumulative.

The snail plot in Figure 2 provides a summary of the assembly statistics, indicating the distribution of scaffold lengths and other assembly metrics. Figure 3 shows the distribution of scaffolds by GC proportion and coverage. Figure 4 presents a cumulative assembly plot, with separate curves representing different scaffold subsets assigned to various phyla, illustrating the completeness of the assembly.

**Figure 5.**
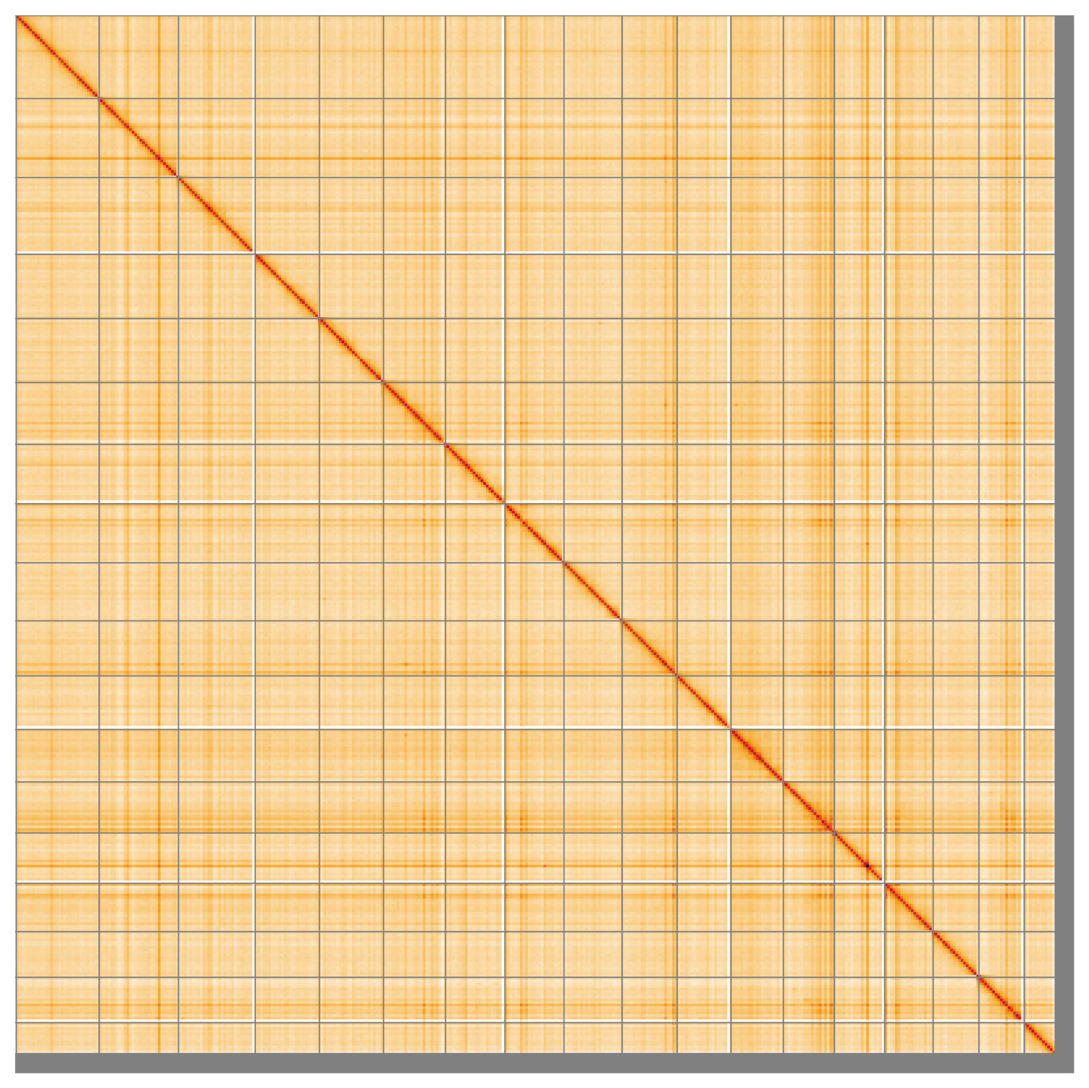
Genome assembly of *Ctena decussata*: Hi-C contact map of the xbCteDecu1.1 assembly, visualised using HiGlass. Chromosomes are shown in order of size from left to right and top to bottom. An interactive version of this figure may be viewed at https://genome-note-higlass.tol.sanger.ac.uk/l/?d=GrdP6hnGSwCrVMvfp76jkQ.

Most of the assembly sequence (97.84%) was assigned to 18 chromosomal-level scaffolds. These chromosome-level scaffolds, confirmed by Hi-C data, are named according to size ( Figure 5; Table 3).

**Table 3.** Chromosomal pseudomolecules in the genome assembly of *Ctena decussata*, xbCteDecu1.

INSDC accession	Name	Length (Mb)	GC%
OZ022498.1	1	129.49	38
OZ022499.1	2	123.76	38
OZ022500.1	3	119.85	38
OZ022501.1	4	100.32	38
OZ022502.1	5	99.98	38
OZ022503.1	6	96.78	38
OZ022504.1	7	92.74	38
OZ022505.1	8	92.3	38
OZ022506.1	9	90.71	38
OZ022507.1	10	85.97	38
OZ022508.1	11	84.08	38
OZ022509.1	12	81.75	38.5
OZ022510.1	13	79.66	38
OZ022511.1	14	78.6	38
OZ022512.1	15	75.65	38
OZ022513.1	16	71.4	38
OZ022514.1	17	70.74	38
OZ022515.1	18	48.41	38
OZ022516.1	MT	0.05	42

The mitochondrial genome was also assembled. This sequence is included as a contig in the multifasta file of the genome submission and as a standalone record in GenBank.

### Assembly quality metrics

The estimated Quality Value (QV) and *k*-mer completeness metrics, along with BUSCO completeness scores, were calculated for each haplotype and the combined assembly. The QV reflects the base-level accuracy of the assembly, while *k*-mer completeness indicates the proportion of expected *k*-mers identified in the assembly. BUSCO scores provide a measure of completeness based on benchmarking universal single-copy orthologues.

The combined primary and alternate assemblies achieve an estimated QV of 63.4. The *k*-mer completeness is 56.97% for the primary haplotype and 57.87% for the alternate haplotype; and 98.57% for the combined primary and alternate assemblies. BUSCO v.5.5.0 analysis using the mollusca_odb10 reference set ( *n* = 5,295) identified 80.6% of the expected gene set (single = 78.5%, duplicated = 2.0%).

## Metagenome report

**Figure 6.**
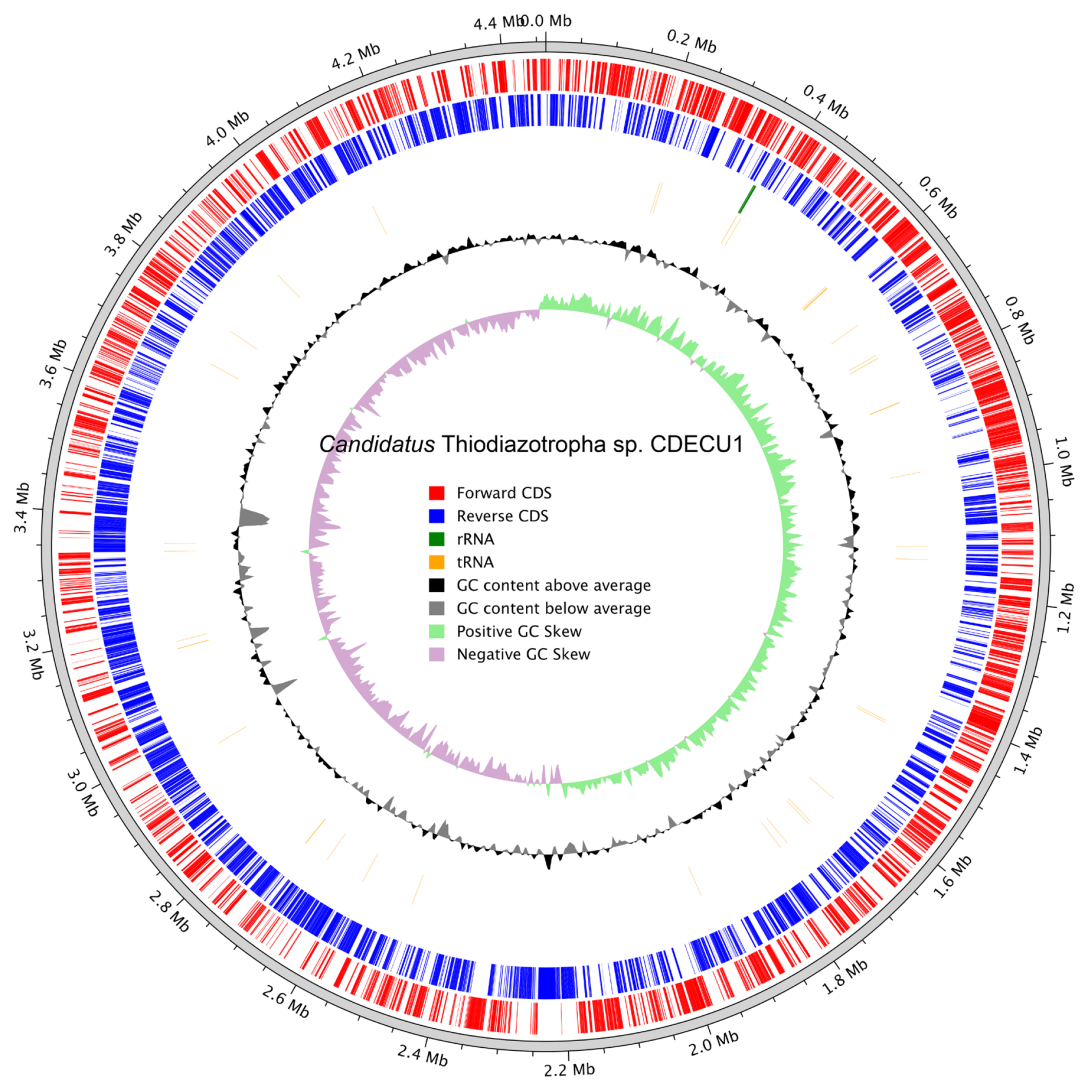
Circular genome map of *Candidatus* Thiodiazotropha sp. CDECU1 (GCA_963455295.1). The outermost rings display annotated forward (red) and reverse (blue) coding sequences (CDSs), ribosomal RNAs (green), and transfer RNAs (orange). The inner black plot shows GC content, with upward and downward deviations from the average. The innermost ring shows GC skew, with green and purple indicating positive and negative skew, respectively.

The genome of the cobiont bacterium *Candidatus* Thiodiazotropha sp. CDECU1 (GCA_963455295.1) was also isolated from the PacBio HiFi reads. The circular bacterial genome is 4.5 Mb in length ( Figure 6).

## Methods

### Sample acquisition

A *Ctena decussata* specimen (specimen no. VIEM1050001, ToLID xbCteDecu1) was collected at 4 m depth in the bay of Sant'Andrea, on the Island of Elba, Italy (latitude 42.81, longitude 10.14) on 2021-02-20. The specimen was taken from its habitat of medium- to coarse-grained silicate sediments around patches of the seagrass *Posidonia oceanica* by Laetitia Wilkins and Miriam Weber (HYDRA Marine Sciences) by digging through the sediment. The specimen was identified by Benedict Yuen based on gross morphology. The specimen was maintained in an aquarium before it was dissected and preserved on 2021-09-06. It was dissected on a mixture of dry ice and ethanol, then frozen in liquid nitrogen, and finally transferred to a –80°C freezer. A second specimen of *Ctena decussata* collected in the same manner was used for RNA sequencing (specimen ID VIEM1050002, ToLID xbCteDecu2).

### Nucleic acid extraction

The workflow for high molecular weight (HMW) DNA extraction at the Wellcome Sanger Institute (WSI) Tree of Life Core Laboratory includes a sequence of procedures: sample preparation and homogenisation, DNA extraction, fragmentation and purification. Detailed protocols are available on protocols.io ( Denton *et al.*, 2023b). The xbCteDecu1 sample was prepared for DNA extraction by weighing and dissecting it on dry ice ( Jay *et al.*, 2023). Tissue from the gill was homogenised using a PowerMasher II tissue disruptor ( Denton *et al.*, 2023a). HMW DNA was extracted using the Automated MagAttract v2 protocol ( Oatley *et al.*, 2023a). DNA was sheared into an average fragment size of 12–20 kb in a Megaruptor 3 system ( Bates *et al.*, 2023). Sheared DNA was purified by solid-phase reversible immobilisation, using AMPure PB beads to eliminate shorter fragments and concentrate the DNA ( Oatley *et al.*, 2023b). The concentration of the sheared and purified DNA was assessed using a Nanodrop spectrophotometer and Qubit Fluorometer using the Qubit dsDNA High Sensitivity Assay kit. Fragment size distribution was evaluated by running the sample on the FemtoPulse system.

RNA was extracted from gill tissue of xbCteDecu2 in the Tree of Life Laboratory at the WSI using the RNA Extraction: Automated MagMax™ *mir*Vana protocol ( do Amaral *et al.*, 2023). The RNA concentration was assessed using a Nanodrop spectrophotometer and a Qubit Fluorometer using the Qubit RNA Broad-Range Assay kit. Analysis of the integrity of the RNA was done using the Agilent RNA 6000 Pico Kit and Eukaryotic Total RNA assay.

### Sequencing

Pacific Biosciences HiFi circular consensus DNA sequencing libraries were constructed according to the manufacturers’ instructions. DNA sequencing was performed by the Scientific Operations core at the WSI on Pacific Biosciences Revio. Tissue from the muscle of the xbCteDecu1 sample was processed for Hi-C sequencing at the WSI Scientific Operations core, using the Arima-HiC v2 kit and sequenced on the Illumina NovaSeq X instrument. Poly(A) RNA-Seq libraries were constructed using the NEB Ultra II RNA Library Prep kit, following the manufacturer’s instructions. RNA sequencing was performed on the Illumina NovaSeq X instrument.

### Genome assembly, curation and evaluation

***Assembly***

Prior to assembly of the PacBio HiFi reads, a database of *k*-mer counts ( *k* = 31) was generated from the filtered reads using FastK. GenomeScope2 ( Ranallo-Benavidez *et al.*, 2020) was used to analyse the *k*-mer frequency distributions, providing estimates of genome size, heterozygosity, and repeat content.

The HiFi reads were assembled using Hifiasm ( Cheng *et al.*, 2021) with the --primary option. Haplotypic duplications were identified and removed using purge_dups ( Guan *et al.*, 2020). The Hi-C reads were mapped to the primary contigs using bwa-mem2 ( Vasimuddin *et al.*, 2019). The contigs were further scaffolded using the provided Hi-C data ( Rao *et al.*, 2014) in YaHS ( Zhou *et al.*, 2023) using the --break option for handling potential misassemblies. The scaffolded assemblies were evaluated using Gfastats ( Formenti *et al.*, 2022), BUSCO ( Manni *et al.*, 2021) and MERQURY.FK ( Rhie *et al.*, 2020).

The mitochondrial genome was assembled using MitoHiFi ( Uliano-Silva *et al.*, 2023), which runs MitoFinder ( Allio *et al.*, 2020) and uses these annotations to select the final mitochondrial contig and to ensure the general quality of the sequence.

***Assembly curation***

The assembly was decontaminated using the Assembly Screen for Cobionts and Contaminants (ASCC) pipeline. Flat files and maps used in curation were generated via the TreeVal pipeline ( Pointon *et al.*, 2023). Manual curation was conducted primarily in PretextView ( Harry, 2022) and HiGlass ( Kerpedjiev *et al.*, 2018), with additional insights provided by JBrowse2 ( Diesh *et al.*, 2023). Scaffolds were visually inspected and corrected as described by Howe *et al*. (2021). Any identified contamination, missed joins, and mis-joins were amended, and duplicate sequences were tagged and removed. The curation process is documented at https://gitlab.com/wtsi-grit/rapid-curation.

***Assembly quality assessment***

The Merqury.FK tool ( Rhie *et al.*, 2020), run in a Singularity container ( Kurtzer *et al.*, 2017), was used to evaluate *k*-mer completeness and assembly quality for the primary and alternate haplotypes using the *k*-mer databases ( *k* = 31) that were computed prior to genome assembly. The analysis outputs included assembly QV scores and completeness statistics.

A Hi-C contact map was produced for the final version of the assembly. The Hi-C reads were aligned using bwa-mem2 ( Vasimuddin *et al.*, 2019) and the alignment files were combined using SAMtools ( Danecek *et al.*, 2021). The Hi-C alignments were converted into a contact map using BEDTools ( Quinlan & Hall, 2010) and the Cooler tool suite ( Abdennur & Mirny, 2020). The contact map is visualised in HiGlass ( Kerpedjiev *et al.*, 2018).

The blobtoolkit pipeline is a Nextflow port of the previous Snakemake Blobtoolkit pipeline ( Challis *et al.*, 2020). It aligns the PacBio reads in SAMtools and minimap2 ( Li, 2018) and generates coverage tracks for regions of fixed size. In parallel, it queries the GoaT database ( Challis *et al.*, 2023) to identify all matching BUSCO lineages to run BUSCO ( Manni *et al.*, 2021). For the three domain-level BUSCO lineages, the pipeline aligns the BUSCO genes to the UniProt Reference Proteomes database ( Bateman *et al.*, 2023) with DIAMOND blastp ( Buchfink *et al.*, 2021). The genome is also divided into chunks according to the density of the BUSCO genes from the closest taxonomic lineage, and each chunk is aligned to the UniProt Reference Proteomes database using DIAMOND blastx. Genome sequences without a hit are chunked using seqtk and aligned to the NT database with blastn ( Altschul *et al.*, 1990). The blobtools suite combines all these outputs into a blobdir for visualisation.

The blobtoolkit pipeline was developed using nf-core tooling ( Ewels *et al.*, 2020) and MultiQC ( Ewels *et al.*, 2016), relying on the Conda package manager, the Bioconda initiative ( Grüning *et al.*, 2018), the Biocontainers infrastructure ( da Veiga Leprevost *et al.*, 2017), as well as the Docker ( Merkel, 2014) and Singularity ( Kurtzer *et al.*, 2017) containerisation solutions.

Table 4 contains a list of relevant software tool versions and sources.

**Table 4.** Software tools: versions and sources.

Software tool	Version	Source
BEDTools	2.30.0	https://github.com/arq5x/bedtools2
BLAST	2.14.0	ftp://ftp.ncbi.nlm.nih.gov/blast/executables/blast+/
BlobToolKit	4.3.9	https://github.com/blobtoolkit/blobtoolkit
BUSCO	5.5.0	https://gitlab.com/ezlab/busco
bwa-mem2	2.2.1	https://github.com/bwa-mem2/bwa-mem2
Cooler	0.8.11	https://github.com/open2c/cooler
DIAMOND	2.1.8	https://github.com/bbuchfink/diamond
fasta_windows	0.2.4	https://github.com/tolkit/fasta_windows
FastK	427104ea91c78c3b8b8b49f1a7d6bbeaa869ba1c	https://github.com/thegenemyers/FASTK
Gfastats	1.3.6	https://github.com/vgl-hub/gfastats
GoaT CLI	0.2.5	https://github.com/genomehubs/goat-cli
Hifiasm	0.19.5-r587	https://github.com/chhylp123/hifiasm
HiGlass	44086069ee7d4d3f6f3f0012569789ec138f42b84aa44357826c0b6753eb28de	https://github.com/higlass/higlass
MerquryFK	d00d98157618f4e8d1a9190026b19b471055b22e	https://github.com/thegenemyers/MERQURY.FK
Minimap2	2.24-r1122	https://github.com/lh3/minimap2
MitoHiFi	2	https://github.com/marcelauliano/MitoHiFi
MultiQC	1.14, 1.17, and 1.18	https://github.com/MultiQC/MultiQC
Nextflow	23.04.1	https://github.com/nextflow-io/nextflow
PretextView	0.2.5	https://github.com/sanger-tol/PretextView
PROKKA	1.14.5	https://github.com/vdejager/prokka
purge_dups	1.2.3	https://github.com/dfguan/purge_dups
samtools	1.19.2	https://github.com/samtools/samtools
sanger-tol/ascc	-	https://github.com/sanger-tol/ascc
sanger-tol/blobtoolkit	0.4.0	https://github.com/sanger-tol/blobtoolkit
Seqtk	1.3	https://github.com/lh3/seqtk
Singularity	3.9.0	https://github.com/sylabs/singularity
TreeVal	1.2.0	https://github.com/sanger-tol/treeval
YaHS	1.1a.2	https://github.com/c-zhou/yahs

## Metagenome assembly

The bacterial cobiont assembly was generated using metaMDBG ( Benoit *et al.*, 2024), binned using MetaBAT2 ( Kang *et al.*, 2019) and optimised using DAS Tool ( Sieber *et al.*, 2018). PROKKA ( Seemann, 2014) was used to identify tRNAs and rRNAs, CheckM (checkM_DB release 2015-01-16) was used to assess bin completeness/contamination, and GTDB-TK (GTDB release 214) ( Chaumeil *et al.*, 2022) was used for taxonomic classification.

### Wellcome Sanger Institute – Legal and Governance

The materials that have contributed to this genome note have been supplied by a Tree of Life collaborator. The Wellcome Sanger Institute employs a process whereby due diligence is carried out proportionate to the nature of the materials themselves, and the circumstances under which they have been/are to be collected and provided for use. The purpose of this is to address and mitigate any potential legal and/or ethical implications of receipt and use of the materials as part of the research project, and to ensure that in doing so we align with best practice wherever possible. The overarching areas of consideration are:

•     Ethical review of provenance and sourcing of the material

•     Legality of collection, transfer and use (national and international)

Each transfer of samples is undertaken according to a Research Collaboration Agreement or Material Transfer Agreement entered into by the Tree of Life collaborator, Genome Research Limited (operating as the Wellcome Sanger Institute) and in some circumstances other Tree of Life collaborators.

## Data Availability

European Nucleotide Archive: Ctena decussata. Accession number PRJEB65003; https://identifiers.org/ena.embl/PRJEB65003. The genome sequence is released openly for reuse. The *Ctena decussata* genome sequencing initiative is part of the Aquatic Symbiosis Genomics (ASG) project ( https://www.ebi.ac.uk/ena/browser/view/PRJEB43743). All raw sequence data and the assembly have been deposited in INSDC databases. The genome will be annotated using available RNA-Seq data and presented through the Ensembl pipeline at the European Bioinformatics Institute. Raw data and assembly accession identifiers are reported in Table 1 and Table 2.

## Author information

Members of the Wellcome Sanger Institute Tree of Life Management, Samples and Laboratory Team are listed here: https://doi.org/10.5281/zenodo.13960213.

Members of the Wellcome Sanger Institute Scientific Operations: Sequencing Operations are listed here: https://doi.org/10.5281/zenodo.14870789.

Members of the Wellcome Sanger Institute Tree of Life Core Informatics team are listed here: https://doi.org/10.5281/zenodo.15495178.

Members of the European Bioinformatics Institute ASG Data Portal team are listed here: https://doi.org/10.5281/zenodo.10076466.

Members of the Wellcome Sanger Institute/Aquatic Symbiosis Genomics Project Leadership are listed here: https://doi.org/10.5281/zenodo.10184833.
