# Supplementary material for: The chromosomal genome sequence of the mollusc, Ctena decussata (O.G.Costa, 1829) and its bacterial endosymbiont Candidatus Thiodiazotropha sp. CDECU1 (Chromatiales)
Source: Wellcome Open Res. 2025 Aug 11;10:435. [Version 1] doi: 10.12688/wellcomeopenres.24738.1 (PMC12485333; doi:10.12688/wellcomeopenres.24738.1)
Supplement: Peer review history

# Reviewer report: Reviewer response for version 1

**DOI:** 10.21956/wellcomeopenres.27255.r130793

**Published:** 2025-11-27

**Authors:** Parin Jirapatrasilp

**Affiliations:** 1 Chulalongkorn University, Bangkok, Bangkok, Thailand

My review is as follows:

 1) The abstract is too short, more detail on bacterium genome could be given in the abstract.

 2) Please provide the full term for the abbreviation mentioned for the first time (e.g,, ANI)

 3) In "Sequencing data" section, "Chromosome conformation Hi-C data produced 0.00 Gb from 0.00 million reads.", I think the numbers are missing here.

 4) Please provide the summary of data retrieved from RNA sequencing in this section.

 5) Please provide a brief interpretation or significant finding based on Fig. 3 and Fig. 4.

 6) Are there no details regarding the mitochondrial genome assembled in this study?

 7) Is there no number summary (e.g., no. of CDS, rRNAs, tRNAs, GC content, etc.) regarding the genome of the cobiont bacterium Candidatus Thiodiazotropha sp. CDECU1?

Are sufficient details of methods and materials provided to allow replication by others?

Yes

Is the rationale for creating the dataset(s) clearly described?

Yes

Are the datasets clearly presented in a useable and accessible format?

Yes

Are the protocols appropriate and is the work technically sound?

Yes

Reviewer Expertise:

Mollusc genomics

I confirm that I have read this submission and believe that I have an appropriate level of expertise to confirm that it is of an acceptable scientific standard.

# Reviewer report: Reviewer response for version 1

**DOI:** 10.21956/wellcomeopenres.27255.r130792

**Published:** 2025-09-30

**Authors:** Markus Pfenninger

**Affiliations:** 1 Senckenberg Biodiversity and Climate Research Centre, Frankfurt am Main, Germany

The Data note of Wilkins et al. describes the genome sequencing of a marine bivalve distributed in the Eastern Atlantik and the Mediterranean, including the genome of an associated bacterium. The article is well structured, the methods clear and, as far as I can tell, up to date.

 The genome was assembled to chromosome scale with largely convincing quality metrics. The only thing I stumbled upon was the sentence *"Chromosome conformation Hi-C data produced 0.00 Gb from 0.00 million reads"*, which is even more strange, because further down, it is assured that these obviously non-existing data was used confirm the chromosome level scaffolds. Please clarify.

Are sufficient details of methods and materials provided to allow replication by others?

Yes

Is the rationale for creating the dataset(s) clearly described?

Yes

Are the datasets clearly presented in a useable and accessible format?

Yes

Are the protocols appropriate and is the work technically sound?

Yes

Reviewer Expertise:

Population genomics, molecular ecology

I confirm that I have read this submission and believe that I have an appropriate level of expertise to confirm that it is of an acceptable scientific standard.

# Reviewer report: Reviewer response for version 1

**DOI:** 10.21956/wellcomeopenres.27255.r130796

**Published:** 2025-09-18

**Authors:** Kyle E McElroy

**Affiliations:** 1 Department of Ecology, Evolutionary, and Organismal Biology, Iowa State University, Ames, Iowa, USA

**Summary**

 The authors report details on the assembly of a chromosome-level genome assembly for a species of bivalve, *Ctena decussata*. This species belongs to family Lucinidae, which is notable for its species’ associations with chemosynthetic endosymbiotic bacteria. Therefore, this report also includes a complete genome for the symbiont, *Candidatus *Thiodiazotropha sp. CDECU1. The genome assembly for *Ctena decussata* appears to be of high quality and completeness, as indicated by its chromosome resolution and its BUSCO and QV scores. I think the methods and results are clear and sufficiently detailed. I only have a few minor comments.

**Minor comments**

 “These resources will also enable further studies investigating the genomic basis of the unique morphological, physiological, and behavioral adaptations that have accompanied the emergence of chemosymbiosis across different animal groups.” – An example of the types of unique adaptations to accompany such symbiotic relationships known to this lineage or others would be useful here.

 I think a brief description of the Hi-C protocol in *Nucleic acid extraction* would be useful. Right now, there is only a mention of the kit used in *Sequencing*.

 There is mention of using GenomeScope2 to estimate genome size and genome-wide heterozygosity from reads. The method for k-mer counting was not described, though I imagine it was Jellyfish. This detail should be added in. Furthermore, the results from this analysis appear to be missing from this report. I do not see a figure or textual description.

 Similarly, I do not see any results for the mitochondrial genome annotation with MitoFinder (which should also be included in Table 4). Also, was the assembled mitogenome a circular molecule? I would say that 53Kb is large for an animal mitogenome, how does it compare to other Lucinidae?

 Regarding the **Metagenome report**, how “complete” is it terms of gene content and how does it compare to previous assembled genomes (i.e., the ones mentioned in Background)?

**Other specific comments**

- “ANI” is not defined in **Background**.
- The Calvin-Benson-Bassham pathway should be defined in **Background**, and its significance noted.
- “Chromosome conformation Hi-C data produced 0.00 Gb from 0.00 million reads”: this text from *Sequencing data* in **Genome sequence report** seems to be missing the specific information from this project.
- Some occasional typos or missing words, for example “50 coverage of the genome” should probably be “50X coverage of the genome”.
- “and maps used in curation were generated via the TreeVal pipe” – this text was bold in the PFD I downloaded. I do not think that it was intended to be.
- In Table 2, the *k*-mer completeness is lower than listed under the “Benchmark” value, is there any reason to be concerned with this result?
- Was a second specimen used for the RNA sample because these are so small?
- I think for these reports showing (or at least reporting in text) the Metazoa BUSCO results will tend to reflect the quality of the assembly better. I checked on the blobtoolkit genomehubs, the metazoan score for this assembly is 93.3% vs. 80.6% for mollusca, which might give readers a sense that this genome is incomplete or the species has had major gene loss. Most mollusks have low scores from the molluca database.

Are sufficient details of methods and materials provided to allow replication by others?

Yes

Is the rationale for creating the dataset(s) clearly described?

Yes

Are the datasets clearly presented in a useable and accessible format?

Yes

Are the protocols appropriate and is the work technically sound?

Yes

Reviewer Expertise:

Molecular and genomic evolution, mollusks, genome assembly and analysis

I confirm that I have read this submission and believe that I have an appropriate level of expertise to confirm that it is of an acceptable scientific standard, however I have significant reservations, as outlined above.

# Reviewer report: Reviewer response for version 1

**DOI:** 10.21956/wellcomeopenres.27255.r130788

**Published:** 2025-09-09

**Authors:** Zohaib Noor

**Affiliations:** 1 University of Chinese Academy of Sciences, Beijing, Beijing, China; 2 Hainan University College of Ocean Sciences (Ringgold ID: 554981), Haikou, Hainan, China

**1. Title and Abstract**

-  Title is clear and informative.
-  Abstract includes essential information (assembly size, chromosomal scaffolds, mitochondrial genome, and symbiont).
- Minor issue: Abstract does not mention assembly quality metrics (BUSCO score, QV), which are important in genome notes.

**2. Background**

- Taxonomy and distribution are well explained.
- Ecological context (symbiosis, metabolic functions) is strong.
- Possible repetition: The Background section re-states some ecological roles in multiple places. Could be shortened.
- Claim: “ *Ctena decussata* is most abundant in the Mediterranean” should be backed with quantitative data (population studies).

**3. Sequencing & Assembly**

-  Sequencing depth (~50× coverage with PacBio HiFi) is solid.
-  Problem: Hi-C sequencing is listed as “0.00 Gb from 0.00 million reads” in Table 1.
  - This seems like either missing data or an error in reporting. Yet later in the text, they state that Hi-C data were used for scaffolding. Needs clarification.
-  Manual curation steps are detailed.
- BUSCO completeness (80.6%) is lower than expected for a reference-grade genome (>90% is standard). Authors should acknowledge this limitation and discuss potential causes (e.g., gene loss, assembly fragmentation).

**4. Symbiont Genome**

- Well described (4.5 Mb circular genome, metabolic capabilities).
- ANI comparison (>96–98% similarity with *C. orbiculata* symbiont) is noted but lacks discussion of functional implications (e.g., whether this suggests species-level identity or just strain-level similarity).

**5. Methods**

- DNA and RNA extraction protocols are clear.
- Software versions are documented in Table 4 (good transparency).
- Some redundancy, e.g., BlobToolKit pipeline description is lengthy and could be summarized.
- Methods mention RNA-Seq data generation, but results do not show transcriptome-supported annotation yet (they say annotation will be added later). This makes the resource incomplete at present.

**6. Figures & Tables**

- Figures (snail plot, blob plot, Hi-C contact map) are appropriate.
- The “Hi-C contact map” figure relies on data, but Table 1 shows no Hi-C reads. Inconsistency again.

**7. Data Availability**

-  ENA accession numbers are provided.
- Genome annotation not yet available. Authors should clearly state when the annotation will be released (timeline).

**8. Overall Strengths**

- Provides the first high-quality genome for *Ctena decussata* and its endosymbiont.
- Transparent in methodology (protocols.io links, software versions).
- Valuable resource for studying chemosymbiosis and bivalve evolution.

**9. Weaknesses / Possible Errors**

1. Hi-C sequencing inconsistency: Table 1 shows “0.00 Gb”, but Hi-C data were used in assembly (contradiction).
2. BUSCO completeness (80.6%) is low and should be acknowledged as a limitation.
3. Repetition in background: Could be more concise.
4. Annotation missing: RNA-Seq was performed, but annotation results have not yet been reported.
5. Symbiont ANI discussion: Needs clarification on taxonomic/functional interpretation.

 
**Conclusion:**

 The genome note is overall strong, but it has inconsistencies (notably the Hi-C data), a lower-than-expected completeness score, and some redundancy in text. Addressing these will make the paper more rigorous and clearer.

Are sufficient details of methods and materials provided to allow replication by others?

Yes

Is the rationale for creating the dataset(s) clearly described?

Yes

Are the datasets clearly presented in a useable and accessible format?

Yes

Are the protocols appropriate and is the work technically sound?

Yes

Reviewer Expertise:

Mollusc breeding, genetics, immunology, and physiological role.

I confirm that I have read this submission and believe that I have an appropriate level of expertise to confirm that it is of an acceptable scientific standard.
